# A correlation analysis between the expression of pregnancy-associated plasma protein A in basal decidual cells and recurrent spontaneous abortion

**DOI:** 10.3892/etm.2013.1149

**Published:** 2013-06-07

**Authors:** YING ZHANG, QIAN ZHAO, YA XIE, KE SU, JUN YANG, LI YANG

**Affiliations:** 1 Department of Gynecology and Obstetrics, The First Affiliated Hospital of Zhengzhou University, Zhengzhou, Henan 450052;; 2 Department of Gynecology and Obstetrics, The First Affiliated Hospital of Xinxiang Medical University, Weihui, Henan 453100;; 3 Department of Gynecology and Obstetrics, The Third Affiliated Hospital of Zhengzhou University, Zhengzhou, Henan 450052, P.R. China

**Keywords:** pregnancy-associated plasma protein A, recurrent spontaneous abortion, basal decidual cells, correlation, prognosis

## Abstract

The aim of this study was to investigate the correlation between the expression of pregnancy-associated plasma protein A (PAPP-A) in basal decidual cells and recurrent spontaneous abortion (RSA). A total of 39 patients with a history of RSA were enrolled into the RSA group. A further 30 females who had experienced normal pregnancy were enrolled into the control group. The mRNA expression of PAPP-A in basal decidual cells was analyzed using real-time PCR. The distribution and expression of PAPP-A protein levels in basal decidual cells were analyzed by immunohistochemistry. The correlation between PAPP-A protein levels and RSA was analyzed. The levels of PAPP-A mRNA in the RSA group were significantly decreased, compared with the control group (P<0.05). Consistent with the mRNA levels, the protein levels of PAPP-A were also significantly lower in the RSA group compared with the control group (P<0.05). Multivariate logistic analysis indicated that the suppression of PAPP-A was one of the risk factors for RSA. Furthermore, Hosmer-Lemeshow analysis suggested that the expression levels of PAPP-A is an important factor for predicting RSA. In conclusion, the expression levels of the PAPP-A protein were significantly reduced in basal decidual cells of the RSA group compared with the control group. Therefore, PAPP-A is likely to play an important role in RSA.

## Introduction

Recurrent spontaneous abortion (RSA) is defined as three or more spontaneous abortions of a fetus before 20 weeks of gestation (1,2). It is one of the most common complications during pregnancy. Common pathogeny including infections, endocrine disorders, heritable mutations and immune deficiencies, have not been identified in RSA (3). Therefore, the most important pathogeny of RSA remains unclear. Pregnancy-associated plasma protein A (PAPP-A) is characterized as plasma glycoprotein secreted by placental syntrophoblastic cells and decidual cells. Previously PAPP-A was shown to be involved in a number of processes during embryonic development, including the early development of gametes, the implantation of zygote and the development of fetus (4). In addition, the expression levels of PAPP-A in placental tissue increases with the time course of pregnancy. In this study, we investigated the correlation between the expression levels of PAPP-A and RSA.

## Subjects and methods

### Subjects

A total of 39 RSA patients from June 2010 to June 2012, termed the RSA group, were enrolled in this study. The criteria for enrolment were: i) Normal cytogenetic phenotype without any heritable disease or spontaneous abortion in the family history; ii) Negative physical examination of vaginal infection; iii) Negative for anticardiolipin antibodies, antinuclear antibodies, antisperm antibodies and antiendometrium antibodies; iv) No autogenous immune disease or endocrine disease; v) No vascular disease or infection disease history; vi) No reproductive disorder or sperm impairment of the fetus’ father; vii) No addiction to cigarettes or alcohol. The age range of the RSA group was 27–41 years, with an average of 33.1±5.4 years. The pregnancy time period was 5–7 weeks, with an average of 6.1±0.8 weeks. In addition to the RSA group, 30 patients who were experiencing normal pregnancy, but who were subjected to induced abortion, were enrolled as a control group. The age range of the control group was 23–40 years, with an average of 32.1±5.2 years. The pregnancy period time was 5–8 weeks, with an average of 6.5±0.9 weeks. No significant difference concerning age and pregnancy time period between the two groups was observed (P>0.05). This study was conducted in accordance with the declaration of Helsinki and with the approval from the Ethics Committee of the Third Affiliated Hospital of Zhengzhou University. Written informed consent was obtained from all the participants.

### qPCR results of PAPP-A mRNA

Basal decidual tissue was obtained using vacuum suction and stored in liquid nitrogen. This tissue was then removed from liquid nitrogen and resolved in 1 ml TRIzol (Invitrogen, Carlsbad, USA). After resolving, 200 *μ*l chloroform was added into the tissue suspension and mixed well. The mixture was then incubated on ice for 5 min and centrifuged at 7342 × g for 15 min. The supernatant liquid was moved to another new tube to which an equal volume of isopropanol was added and mixed well. The tube was incubated on ice for 10 min and centrifuged at 7342 × g for 15 min. Following aspiration of the supernatant, the tissue was washed in 1 ml chilled 75% alcohol. The tube was gently tapped five times, centrifuged at 4828 × g for 5 min and the supernatant was then carefully aspirated. DEPC-treated water was added to resolve the RNA pellet and the total concentration of RNA was measured. RNA was reversed using a reverse transcriptional kit (Takara, Dalian, China). The resulting cDNA was employed in qPCR analysis performed using the primers: PAPP-A, forward: 5′-CTACTTGGATGTTAATGAGC-3′, and reverse: 5′-TCCTGCCAACTCCTCCTCTG-3′; actin, forward: 5′-AGC GGGAAATCGTGCGTGACA-3′, and reverse: 5′-GTGGA CTTGGGAGAGGACTGG-′3. The primers were synthesized by the Shanghai Yingjun Biotechnology Co. Ltd., Shanghai, China. The primers were resolved at a concentration of 10 *μ*M. The reaction system included 10 *μ*l SYBR^®^ Premix Ex TaqTM (Applied Biosystems, Milan, Italy), 0.6 *μ*l forward primer, 0.6 *μ*l reverse primer, 8.8 *μ*l cDNA diluted at a 1:100 ratio. The total volume of 20 *μ*l for each qPCR reaction was added into a 96-well plate and gently centrifuged at 150 × g for 2 min. Following the initial step of 95°C for 5 min, 40 PCR cycles of 95°C for 30 sec and then 60°C for 1 min, were carried out. The expression levels of PAPP-A mRNA were measured according to the 2^−ΔΔCt^ relative quantification analysis method.

### Immunostaining for PAPP-A protein

Basal decidual tissue from the RSA and control groups were paraffin-embedded. Deparaffinization treatment of a 5 *μ*m section slide was performed prior to staining. Subsequently, the slide was preincubated in PBST three times followed by 3% H_2_O_2_-methanol treatment at 95°C for 15 min. The slide was then washed again in PBST three times and blocked with a medium containing 5% normal goat serum for 1 h at room temperature. The slides were then incubated overnight at 4°C with the primary antibodies (Abcam, Cambridge, UK) diluted in blocking medium. On the second day, the slides were rinsed in PBST and the sections incubated for 30 min at room temperature with the secondary antibodies (Santa Cruz Biotechnology, Santa Cruz CA, USA) diluted in blocking medium. Following rinsing of the slides in PBST three times, the slides were incubated in the DAB exposure medium. DAB exposure medium was then washed away and the slides incubated in hematoxylin for 30 sec. The slides were gradient dehydrated and mounted.

### Immunostaining analysis for PAPP-A

The stained sections were analyzed using Motic Med 6.0 digital medicine image software. For each section, 10 views were randomly chosen under a microscope (magnification, ×400). The PAPP-A-positive cells were counted in all 10 views and the percentage of positive cells in the entire population was calculated. Four grades were defined to analyze the results: Negative(−): % of positive cells, <5%; weak positive(+): % of positive cells, 5–25%; medium positive (++): % of positive cells with a range of, 5–25%; positive(+++): % of positive cells was 25–50%; strong positive (++++): % of positive cells, >50%. Quantitative evaluation was achieved using Motic Med 6.0 digital medicine image software.

### Statistical analysis

Data were shown as mean ± SD. For statistical analyses, the Student’s t-test and the Chi-Square test were used as appropriate by using SPSS 13.0 software (SPSS Inc., Chicago, IL, USA). Multivariate non-conditional logistic regression analysis was used to evaluate the correlation between PAPP-A and RSA. A difference at P<0.05 was considered statistically significant.

## Results

### Comparison of the PAPP-A mRNA levels between the RSA and control groups

**Figure 1.**
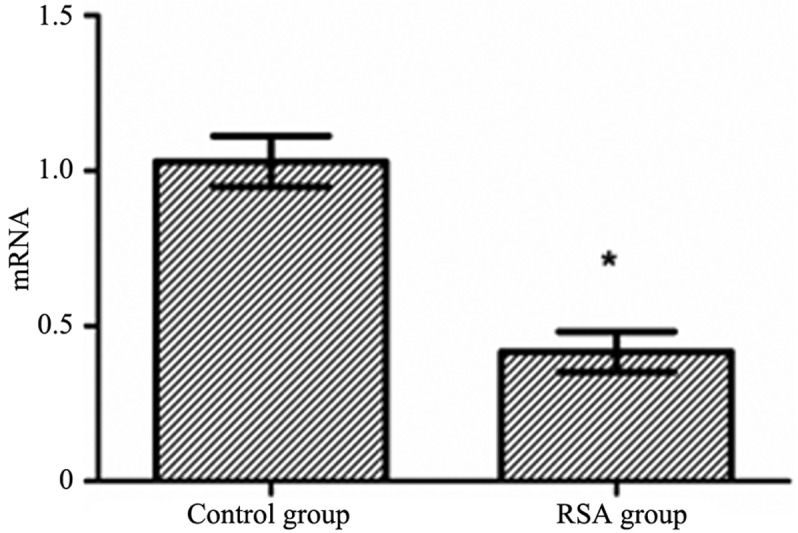
PAPP-A mRNA level of decidual in the RSA and control groups. *P<0.05.

The total RNA in decidual tissue was measured using ultraviolet spectrophotometry. The optical density (OD)260/280 values were in the range of 1.9–2.0. qPCR results demonstrated that the mRNA levels of PAPP-A in the RSA group were significantly decreased compared with the control group (t=5.204, P<0.05) (Fig. 1).

### Comparison of the PAPP-A protein levels between the RSA and control groups

**Figure 2.**
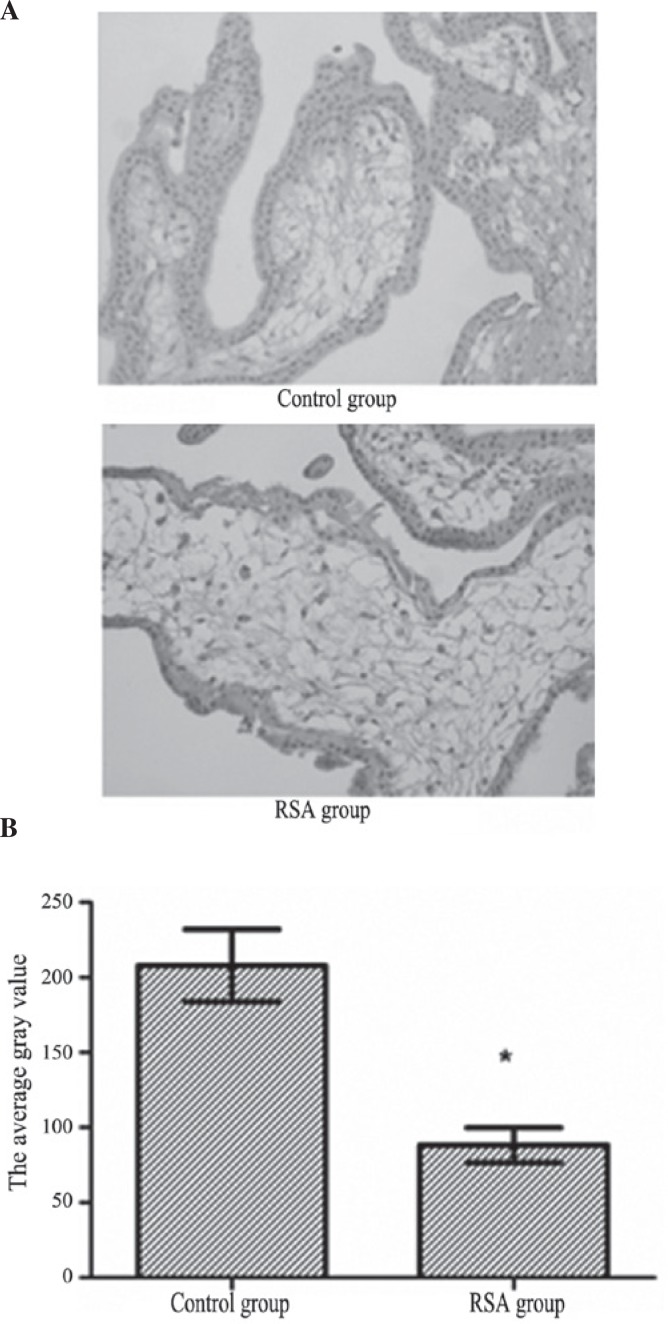
Tissue distribution and quantitative results of the PAPP-A protein between the RSA and control group. ^*^P<0.05.

Immunostaining showed that PAPP-A protein was expressed in the cytoplasm of basal decidual cells (Fig. 2A). The general PAPP-A protein level of the RSA group was weak positive, whereas the general PAPP-A protein level of the control group was strong positive (Table I). The quantitative measurement of PAPP-A protein showed that the protein expression levels were significantly decreased in the RSA group compared with the control group (t=4.464, P<0.05) (Fig. 2B).

### Correlation analysis between the PAPP-A protein level and RSA

The correlation analysis between the PAPP-A protein levels and RSA was evaluated using multivariate logistic analysis. The results showed that the protein expression level of PAPP-A was highly related to RSA, indicating that PAPP-A is one of the major risk factors of RSA. The Hosmer-Lemeshow analysis showed that the protein expression level of PAPP-A is likely a good prognosis reference for RSA (χ^2^=3.158, P<0.05).

## Discussion

The incidence of RSA is approximately 1% in all females experiencing pregnancy. Therefore, it has been a hot topic in clinical and scientific research in recent years (5). Since its complicated pathologic mechanisms include cytogenetic abnormality (6), endocrine disorder (7), immunodeficiency (8), reproductive disease, infection (9), trauma, unhealthy behaviors and environmental factors, the major pathogeny of RSA remains to be determined (10). Previous studies have shown that PAPP-A is abnormally expressed in RSA patients (11,12). In this study, more detailed data that support the correlation between RSA and PAPP-A levels were obtained. The results may provide a further useful prognosis method for this disease.

PAPP-A, which is secreted by placental syntrophoblastic cells and basal decidual cells, is one of the plasma glycoproteins involved in pregnancy. PAPP-A has previously been shown to regulate embryonic development (13,14). It can first be detected in serum during the fifth week of pregnancy, and then increases with time subsequently. Peak PAPP-A expression levels are observed at the end of pregnancy and levels are then downregulated subsequent to delivery (15,16). At present, PAPP-A levels are used as one of the major references for monitoring early pregnancy and evaluating the health of the fetus. During clinical investigations, a low level of PAPP-A expression in RSA patients compared to patients experiencing normal pregnancy has been observed. Similar phenotypes have also been reported by other groups (17). In the present study, the specific transcription and expression levels of PAPP-A were evaluated in an RSA group.

The results have demonstrated that the level of PAPP-A mRNA in basal decidual tissue was significantly decreased in the RSA group patients compared with the control group. To obtain more detailed information on the difference in PAPP-A protein expression level, immunostaining with PAPP-A antibodies was performed. In agreement with the qPCR result, the PAPP-A protein expression levels were also decreased in the RSA group compared with the control group. Furthermore, results of the correlation and Hosmer-Lemeshow analyses suggested that the protein levels of PAPP-A could be used as an important reference in clinical RSA prognosis. The decrease in PAPP-A levels may also be a critical risk factor in RSA.

In conclusion, PAPP-A levels were significantly decreased in RSA patients compared with patients experiencing normal pregnancy. This abnormal expression may be one of the risk factors leading to RSA.

## Figures and Tables

**Table I.** PAPP-A protein expression level in the RSA and control groups.

Group	−	+	++	+++
RSA	2	19	8	1
Control	0	7	11	21

PAPP-A, pregnancy-associated plasma protein A; RSA, recurrent spontaneous abortion.
